# Left atrial function after standalone totally thoracoscopic left atrial appendage exclusion in atrial fibrillation patients with absolute contraindication to oral anticoagulation therapy

**DOI:** 10.3389/fcvm.2022.1036574

**Published:** 2022-11-07

**Authors:** Massimiliano Marini, Luigi Pannone, Stefano Branzoli, Francesca Tedoldi, Giovanni D’Onghia, Diego Fanti, Emanuele Sarao, Fabrizio Guarracini, Silvia Quintarelli, Cinzia Monaco, Angelo Graffigna, Roberto Bonmassari, Mark La Meir, Gian Battista Chierchia, Carlo de Asmundis

**Affiliations:** ^1^Department of Cardiology, S. Chiara Hospital, Trento, Italy; ^2^Heart Rhythm Management Centre, Postgraduate Program in Cardiac Electrophysiology and Pacing, Vrije Universiteit Brussel, Universitair Ziekenhuis Brussel, Brussels, Belgium; ^3^Department of Cardiac Surgery, S. Chiara Hospital, Trento, Italy; ^4^Department of Cardiac Surgery, Vrije Universiteit Brussel, Universitair Ziekenhuis Brussel, Brussels, Belgium

**Keywords:** left atrial appendage exclusion, oral anticoagulation therapy, totally thoracoscopic surgery, atrial fibrillation, left atrial appendage

## Abstract

**Background:**

Left atrial appendage (LAA) is a common source of thrombi in patients with atrial fibrillation (AF). The effect on left atrial (LA) function of the Totally Thoracoscopic (TT)-LAA exclusion with epicardial clip is currently unknown. This study aims at evaluating the effect of TT-LAA exclusion on LA function.

**Methods:**

Standalone TT-LAA exclusion with the clip device was performed in 26 patients with AF and contraindication to oral anticoagulation (OAC). A 3D CT scan, trans-esophageal echocardiography, spirometry and cerebrovascular doppler ultrasound were performed preoperatively. Clip positioning and LAA exclusion were guided and confirmed by intraoperative trans-esophageal echo. To evaluate LA function, standard transthoracic echocardiography and 2D strain of LA were performed before surgery, at discharge and at 3-month follow-up.

**Results:**

The mean CHA_2_DS_2_-VASc and HASBLED scores were 4.6 and 2.4 respectively. There were no major complications during the procedure. At median follow-up of 10.3 months, 1 (3.8%) non-cardiovascular death, 1 (3.8%) stroke and 4 (15.4%) cardiovascular hospitalizations occurred. At 2D strain of LA, the reservoir function decreased significantly at discharge, compared to baseline and recovered at 3-months follow-up. Furthermore, NT-proBNP increased significantly after the procedure with a return to baseline after 3 months. Changes in E/A were persistent at 3 months.

**Conclusion:**

Our data in a small cohort suggest that TT-LAA exclusion with epicardial clip can be a safe procedure with regards to the atrial function. The LAA amputation impairs the reservoir LA function on the short term, that recovers over time.

## Introduction

Atrial fibrillation (AF) is the most common sustained arrhythmia in humans and oral anticoagulation (OAC) to prevent ischemic stroke in AF patients with high CHA_2_DS_2_-VASc risk score is a guideline-recommended therapy (1, 2).

Despite the recent advances in pharmacological stroke prevention the perceived risk of OAC-associated bleeding may result in significant under prescription or under dosage of this therapy (3). The surgical exclusion of the left atrial appendage (LAA) is a therapeutic strategy for stroke prevention in AF patients with an absolute contraindication to OAC or a high risk of life-threatening bleeding on OAC or antiplatelet therapy (APT) (3) and unsuitable for percutaneous LAA occlusion. This intervention effect on left atrial (LA) function has not been studied. The LAA produces vasoactive neuroendocrine hormones activated by stretch-sensitive receptors (4) and this suggests a role in cardiovascular homeostasis as a “decompression chamber.” LAA closure results in an increase LA size and mean pressure from animal models and human studies (5). Recent techniques have been introduced to assess the LA function such as two-dimensional speckle tracking echocardiography (2D STE), and specifically the strain and strain rate parameters. Through these parameters, the three LA function stages (reservoir, conduit and contractile) can be assessed.

This study aims at evaluating the effect of totally thoracoscopic (TT)-LAA exclusion on the LA function, evaluated with 2D STE.

## Materials and methods

### Patient population

This observational and retrospective study enrolled patients with AF at high risk for ischemic stroke and at high risk of life-threatening bleeding on OAC or APT or with a contraindication to long-term OAC. All patients underwent TT-LAA exclusion in the period between March 2020 and June 2021 at S. Chiara Hospital, Trento, Italy.

Inclusion criteria were: (1) AF defined following current guidelines (1); (2) Patients deemed at high risk for ischemic stroke, defined as CHADSVASC > 1 or ≥ 2 if female sex; and (3) contraindication to long term OAC/APT, defined as at least one of the following: HASBLED > 3, previous severe bleeding on OAC/APT or refractory anemia; or (4) refractory LAA thrombosis or recurrent stroke despite different OAC therapies (1). Previous severe bleeding was defined as at least one of the following:, diffuse gastrointestinal hemorrhage requiring transfusions or prior cerebral hemorrhage or other bleeding scenario with BARC > 1 (6).

Final decision on inclusion in the study was taken by the “AF Heart Team,” including a cardiac surgeon, a cardiologist, neurologist/neurosurgeon and referring physician following current guidelines on LAA exclusion (6).

All patients underwent preoperative computed tomography (CT) with 3-dimensional reconstruction, transthoracic and transesophageal echocardiography (TEE) to rule out thrombi in the LAA and to exclude other cardiac surgery indications for structural or functional heart diseases. Spirometry and bilateral carotid ultrasound doppler were also performed during the preoperative work out. Clinical history and laboratory data were collected and analyzed. Patient provided written informed consent to the procedure. The study complied with the Declaration of Helsinki as revised in 2013; the ethic committee approved the study.

### Surgical procedure

All patients were treated using the video assisted thoracoscopic LAA exclusion approach with Atriclip PRO2 device (AtriCure Inc., Mason, OH). The procedure has been previously described in details (7). Briefly, patients were placed in a supine position, selective right lung ventilation was chosen with double lumen ventilation and intraoperative TEE monitoring to evaluate and guide the correct device positioning. Three 12 Fr thoracoscopic access ports were used, including the following: (1) a camera port placed along the mid axillary line at mid sternal level and (2) working ports, placed along the anterior axillary line in the third intercostal space and in the intercostal space at the intersection between the line in the middle of anterior and midaxillary line and a sagittal line crossing the xiphoid process. After insufflation with CO_2_, visualization of the intrathoracic anatomy, and freeing of adhesions, the opening of the pericardium was performed. The LAA was mobilized and the base measured with a dedicated sizer to select the device size. The AtriClip PRO2 (Atri-Cure Inc., West Chester, OH) device was positioned using the dedicated deployment device and deployed under TEE guidance and camera visualization.

### Echocardiographic analysis

Echocardiographic analysis was performed in all patients by experienced cardiologists, a specific protocol was compiled, and the echocardiographic measurements were obtained following current guidelines (8). Standard 2D measurements were performed using GE Vivid E9 or GE Vivid E80, (GE-Healthcare, Chicago, Illinois) and LA deformation was evaluated with a 2D STE software. All images were acquired in a DICOM format and digitally stored for offline analysis. Two different experienced cardiologists performed offline analysis. Echocardiographic parameters analyzed included the following: LV end diastolic volume (EDV), left ventricular (LV) ejection fraction (LVEF), LA volume indexed to body surface area (BSA), mitral peak velocity in early diastole (E) and in late diastole (A), average (mean of septal and lateral) early diastolic mitral annulus velocity (e’) estimated by tissue Doppler. Simpson’s biplane method of discs was used to perform volumetric calculation of both LV and LA. All measurements were performed following ASE guidelines (9).

### Two-dimensional speckle tracking echocardiography

**FIGURE 1:**
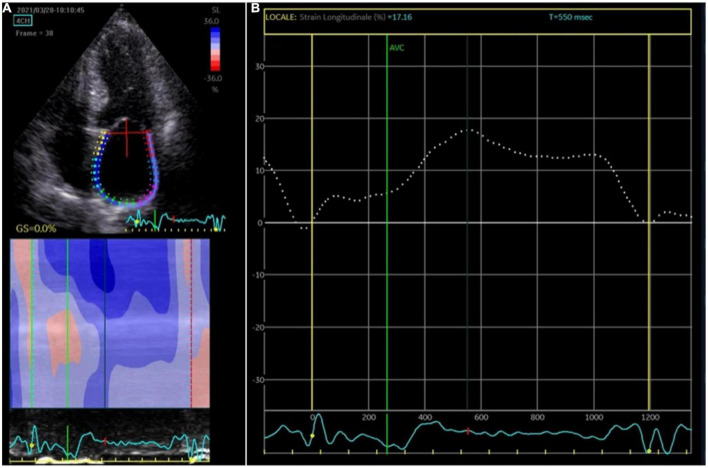
Atrial strain measurement. **(A)** The apical four-chamber view was utilized for the strain measurements of the left atrium (LA); the edge of the LA endocardium was manually traced. **(B)** The software generated tracings based on the 2D strain of LA. The mean deformation (strain) is expressed in percentage and calculated by the software.

Two-dimensional speckle tracking echocardiography was performed with a standard protocol following current guidelines (10). The apical four-chamber view was utilized for the strain measurements of LA and LV. Briefly, first the LA endocardium edge was traced manually and then the tracings based on the 2D STE were generated by the software, Figure 1. The mean deformation (strain) expressed in percentage was then calculated by the software. The reservoir function of the LA (LA reservoir strain) was calculated as the maximal wall deformation of LA during LV systole as compared to the end diastole, that was considered as preset reference point (11). 2D STE LA strain refers to reservoir strain if not otherwise specified. In patients with permanent AF, atrial strain was performed during ongoing AF. In patients with non-permanent AF, atrial strain was performed during sinus rhythm. Global longitudinal strain (GLS) of LV was measured as the longitudinal shortening of the myocardium (change in length compared to the baseline length).

### Follow-up

Periprocedural adverse events were registered. Thoracoscopic access was evaluated after 10 days from the procedure. Clinical evaluations included laboratory work-out at pre-discharge and after 3 months and physical examination. A protocol echocardiogram was performed at baseline (pre-surgery), at pre-discharge (after surgery) and at 3 months. At 3 months follow-up, a cardiac synchronized CT scan or TEE were also performed to measure the size of the residual stump, if any and to assess the efficacy of LAA exclusion. A satisfactory outcome was considered as a residual stump < 1 cm (12). The primary endpoint was LA function, defined with 2D STE at pre-discharge and at 3 months. Secondary endpoints were the following: all-cause mortality, cardiovascular hospitalizations and stroke at long-term follow-up.

### Statistical analysis

Descriptive statistics are reported as medians and interquartile range (IQR) for non-normally distributed continuous variables or mean ± standard deviation for normally distributed continuous variables. *T*-test was used to compare numerical normal variables, and Wilcoxon test for non-parametric variables. The categorical variables were compared by Chi-squared test or Fisher’s exact test and described as frequencies and percentages. A *p*-value < 0.05 was considered significant for all tests. The analysis was performed using R software version 3.6.2 (R Foundation for Statistical Computing, Vienna, Austria).

## Results

### Patient population

The study enrolled 26 consecutive patients (77.3 ± 6 years, 76.9% males). The mean HAS-BLED and CHA_2_DS_2_-VASc scores were 2.4 ± 0.6 and 4.6 ± 1.1 respectively. Permanent AF was present in 8 patients (30.8%). The indication for LAA exclusion was the following: history of cerebral hemorrhage (*n* = 10), diffuse gastrointestinal hemorrhage requiring transfusions (*n* = 4), clinical scenario of high bleeding risk (*n* = 4), refractory anemia (*n* = 3), other bleeding scenarios (*n* = 2), refractory LAA thrombosis (*n* = 1) and recurrent stroke despite different OAC therapies (*n* = 2). Baseline characteristics are summarized in Table 1.

**TABLE 1:** Baseline characteristics.

	Overall (*N* = 26)
Age (years)	77.3 (6.0)
Sex (M) (*n*, %)	20 (76.9%)
BMI	25.6 (4.7)
BSA	1.9 (0.2)
Permanent AF (*n*, %)	8 (30.8%)
CHA2DS2-VASc score	4.6 (1.1)
HASBLED score	2.4 (0.6)
Stroke/TIA history (*n*, %)	6 (23.1%)
Diabetes (*n*, %)	7 (26.9%)
Hypertension (*n*, %)	25 (96.2%)
Heart failure (*n*, %)	9 (34.6%)
CKD (HASBLED definition) (*n*, %)	3 (11.5%)
Peripheral vascular disease (*n*, %)	15 (57.7%)
COPD (*n*, %)	3 (11.5%)
NYHA II–III (*n*, %)	21 (80.8%)
Follow-up (months)	10.3 (4.7)

AF, atrial fibrillation; BMI, body mass index; BSA, body surface area; CKD, chronic kidney disease; COPD, chronic obstructive pulmonary disease; TIA, transient ischemic attack.

### Surgical treatment

A total of 26 patients underwent thoracoscopic LAA exclusion. Mean operation time (skin-to-skin) was 69.2 ± 18.5 min. No deaths procedure-related or pulmonary morbidity were observed. No patient required conversion to mini thoracotomy. Intraoperative TEE showed complete LAA exclusion with minimal residual stump (<1 cm) in all cases. Following the procedure, no patients were prescribed OAC, APT or heparin. There were no major complications during and after the procedure.

### Laboratory and echocardiographic changes

Compared with baseline, NT-proBNP was significantly higher at pre-discharge evaluation (1000.3 ± 950.1 pg/ml vs. 3170.2 ± 2011.6 pg/ml, *p* < 0.001); after 3 months there was no differences in NT-proBNP value compared with baseline (*p* = 0.17) (Table 2). There were no significant differences in creatinine and hemoglobin at pre-discharge and at 3-months follow-up (Table 2).

**TABLE 2:** Laboratory and echocardiographic data during follow-up.

	Baseline (*N* = 26)	Pre-discharge (*N* = 26)	*P*-value (baseline vs. pre-discharge)	3 months follow-up (*N* = 26)	*P*-value (baseline vs. 3 months follow-up)	*P*-value (pre-discharge vs. 3 months follow-up)
NT-proBNP (pg/ml)	1000.3 (950.1)	3170.2 (2011.6)	<0.001	1509.5 (1444.3)	0.17	0.005
Creatinine (mg/dl)	1.4 (0.9)	1.5 (1.1)	0.86	1.7 (1.7)	0.46	0.56
Hb (g/dl)	12.3 (1.5)	12.0 (1.5)	0.37	12.5 (2.2)	0.75	0.34
LVEF (%)	58.9 (8.4)	58.1 (6.5)	0.72	56.3 (9.2)	0.32	0.46
GLS (%)	16.8 (4.5)	14.6 (4.2)	0.11	15.3 (4.2)	0.28	0.59
EDV (ml)	103.7 (27.7)	108.8 (33.2)	0.57	113.8 (27.9)	0.24	0.60
E wave (m/s)	0.8 (0.3)	0.9 (0.2)	0.48	0.8 (0.3)	0.99	0.47
A wave (m/s)	0.89 (0.2)	0.81 (0.3)	0.44	0.61 (0.3)	0.005	0.08
E/A	0.9 (0.3)	1.1 (0.3)	0.25	1.3 (0.4)	0.004	0.07
dT (ms)	165.0 (42.7)	168.0 (49.4)	0.83	183.2 (39.2)	0.16	0.28
E’ (m/s)	0.1 (0.0)	0.1 (0.0)	0.31	0.1 (0.0)	0.17	0.74
E/e’	13.1 (6.8)	12.7 (6.8)	0.85	10.6 (3.0)	0.14	0.20
PAPs	33.7 (11.7)	33.4 (13.5)	0.96	30.6 (9.7)	0.47	0.55
LA volume index	59.3 (33.0)	56.6 (26.9)	0.76	59.2 (28.3)	0.99	0.76
LA reservoir strain	16.9 (7.7)	11.8 (8.0)	0.028	18.6 (10.5)	0.55	0.017

dT, deceleration time; EDV, end-diastolic volume; GLS, global longitudinal strain; Hb, hemoglobin; LA, left atrium; LVEF, left ventricular ejection fraction; PAPs, systolic pulmonary artery pressure.

The 2D STE of LA measured at pre-discharge decreased significantly compared with the baseline values (11.8 ± 8% vs. 16.9 ± 7.7%, *p* = 0.028) with a recovery at 3-months (18.6 ± 10.5% vs. 16.9 ± 7.7%, *p* = 0.55) (Table 2). When compared with baseline, E/A increased significantly after 3 months (1.3 ± 0.4 vs. 0.9 ± 0.3, p = 0.004) (Table 2). Of note, there was a non-significant trend toward higher E/A values at pre-discharge evaluation compared to baseline (1.1 ± 0.3 vs. 0.9 ± 0.3, p = 0.25). E/e’ decreased throughout serial evaluation with no significant change (13.1 ± 6.8 vs. 12.7 ± 6.8 vs. 10.6 ± 3.0, *p* = NS for all comparisons). At pre-discharge and at 3-months follow-up echocardiography, the LA volume indexed to BSA was unchanged compared with baseline measurements (56.6 ± 26.9 ml/mq vs. 59.2 ± 28.3 ml/mq vs. 59.3 ± 33.0 ml/mq, *p* = NS for all comparisons). There was no significant difference in LV EDV, LVEF and GLS of LV (Table 2). The results of 2D STE of LA for permanent AF patients compared with non-permanent AF patients are summarized in Table 3.

**TABLE 3:** 2D strain results for permanent vs. non-permanent atrial fibrillation patients.

	Non-permanent AF (*N* = 18)	Permanent AF (*N* = 8)	Overall (*N* = 26)	*P*-value
LA reservoir strain baseline	19.9 (7.0)	10.1 (4.2)	16.9 (7.7)	0.003
LA reservoir strain pre-discharge	14.2 (8.6)	6.6 (2.2)	11.8 (8.0)	0.023
LA reservoir strain 3 months follow-up	20.5 (9.9)	11.0 (10.7)	18.6 (10.5)	0.11

AF, atrial fibrillation; LA, left atrium.

### Follow-up

Follow up was completed and available for all 26 patients. At a median follow-up of 10.3 ± 4.7 months, no patients were on OAC, APT or heparin therapy. One (3.8%) non-cardiovascular death, 1 (3.8%) stroke and 4 (15.4%) cardiovascular hospitalizations occurred at long-term follow up. Evaluation by TEE or CT after 3 months showed stable and appropriate device position with LAA stump < 1 cm in all patients.

## Discussion

The main findings of this study can be summarized as follows: (1) The amputation of LAA significantly impairs LA reservoir function after the procedure, although this function recovers after 3 months. (2) TT-LAA exclusion results in a change in E/A that is persistent at 3 months follow-up.

### The role of left atrial appendage clipping on left atrial reservoir function

The LA function consists of three components, namely: conduit, reservoir and pump. It is the result of a complex interplay between LV systolic and diastolic function, circulating blood volume and LAA function (13).

In the current study a transient impairment of LA reservoir function was observed after LAA exclusion; different mechanisms might contribute to this finding.

The sudden volume reduction of the LA after the procedure may affect LA distension, whereas its recovery might be explained with volume recovery or LA remodeling over time.

Changes in LA function could also be secondary to an altered neuro-humoral homeostasis expressed by changes in both atrial natriuretic peptide (ANP) and NT-proBNP. LAA endovascular occlusion is associated with an increase in ANP levels (14); ANP is produced by LAA and it plays an important role in LA physiology (15); its mutation is associated with a familial atrial dilated cardiomyopathy with standstill evolution (15). In a previous study on endovascular LAA closure, NT-proBNP was higher at 6 h and 24 h after procedure with no difference at 48 h (16). This is consistent with our results of a sudden increase in NT-proBNP followed by a return to baseline values.

Previous studies with percutaneous LAA closure, were characterized by heterogeneous results; different groups showed no changes in LA function after the procedure (17–19). A limitation of previous studies was the lack of routine 2D STE. Indeed, a subtle difference in LA function was evident only at 2D STE in our study. Furthermore, other groups demonstrated an improvement in LA function (20). The technical difference between the percutaneous and the surgical approach (with the latter causing a clean anatomical exclusion of the LAA) could explain the different behavior of the atrial function during the follow-up (21). In patients undergoing TT pulmonary veins isolation and LAA exclusion: De Maat et al. (21) concluded that the LAA exclusion does not impair the LA contractile function or the ejection fraction of LA, but there is only a reduction in LA reservoir function, in contrast Gelsomino et al. (22) described a gain of LA function and a reverse LA remodeling after the surgical ablation and LAA exclusion.

The recovery of reservoir strain of LA after 3 months is consistent with previous studies with 2D STE (20, 23); it might be explained by the recovery of both LA preload and neuro-humoral homeostasis. LAA exclusion might improve mechanical function of the LA and result in reverse LA remodeling (24).

Pulsed wave measures, in particular E/A remained increased at 3 months follow-up in the current study. In the pulsed wave analysis of transmitral flow, E wave represents the early fast diastolic filling and it is a measure of LA reservoir function. Its increase, with a consequent increase of E/A has been reported in previous studies (25); it could be a consequence of LA volume reduction following LAA exclusion, that represents the most distensible portion of LA (26).

## Limitations

The main limitation of the study is that it is retrospective. The included number of patients was relatively small, due to strict inclusion criteria. Limitations also included referral bias, being the center specialized in TT treatment of AF and TT-LAA exclusion. The reported changes in reservoir function might depend also on the appendage volume, which may differ among individuals. Data on LAA volume are lacking in all the published studies, and in the present one. In patients with permanent AF, A wave was not measured.

## Conclusion

Our data in a small cohort suggest that TT-LAA exclusion with epicardial clip can be a safe procedure with regards to the atrial function. The LAA amputation impairs the reservoir LA function on the short term, that recovers over time.

## Data availability statement

The raw data supporting the conclusions of this article will be made available by the authors, without undue reservation.

## Ethics statement

The studies involving human participants were reviewed and approved by the Santa Chiara Hospital Ethics Committee. The patients/participants provided their written informed consent to participate in this study.

## Author contributions

MMa, LP, and CA: conception and design of the work. MMa, LP, SB, FT, GD’O, DF, and ES: substantial contributions to the acquisition of data for the work. MMa and LP: substantial contributions to the analysis of data for the work and drafting the work. AG, RB, MMe, GC, and CA: substantial contributions to the interpretation of data for the work. FG, SQ, CM, AG, RB, MMe, GC, and CA: revising the draft of the work critically for important intellectual content. MMa, LP, SB, FT, GD’O, DF, ES, FG, SQ, CM, AG, RB, MMe, GC, and CA: final approval of the version to be published and agreement to be accountable for all aspects of the work in ensuring that questions related to the accuracy or integrity of any part of the work are appropriately investigated and resolved. All authors contributed to the article and approved the submitted version.
